# Pathway for Biodegrading Nodularin (NOD) by *Sphingopyxis* sp. USTB-05

**DOI:** 10.3390/toxins8050116

**Published:** 2016-05-04

**Authors:** Nan Feng, Fan Yang, Hai Yan, Chunhua Yin, Xiaolu Liu, Haiyang Zhang, Qianqian Xu, Le Lv, Huasheng Wang

**Affiliations:** 1 School of Chemistry and Biological Engineering, University of Science and Technology Beijing, Beijing 100083, China; ustbnanfeng@163.com (N.F.); ustbyangfan@163.com (F.Y.); chyin@ustb.edu.cn (C.Y.); xiaoluliu@ustb.edu.cn (X.L.); zhanghy@ustb.edu.cn (H.Z.); qianqianxu@ustb.edu.cn (Q.X.); lvle@ustb.edu.cn (L.L.); 2 School of Architectural and Surveying & Mapping Engineering, Jiangxi University of Science and Technology, Ganzhou 341000, China; wanghuasheng2005@126.com

**Keywords:** nodularin (NOD), *Sphingopyxis* sp. USTB-05, crude enzymes (CEs), biodegradation pathway

## Abstract

Nodularin (NOD) is greatly produced by *Nodularia spumigena* and released into the environment when toxic cyanobacterial blooms happened in natural water body, which is seriously harmful to human and animals. The promising bacterial strain of *Sphingopyxis* sp. USTB-05 was found to have an ability in biodegrading NOD. Initially, 11.6 mg/L of NOD could be completely eliminated within 72 h by whole cells of USTB-05, and within 36 h by its crude enzymes (CEs) of 570 mg/L, respectively. During the enzymatic biodegradation process of NOD, two products were observed on the profiles of HPLC. Based on the analysis of *m*/*z* ratios of NOD and its two products on a rapid-resolution liquid chromatogram-mass spectrum (RRLC-MS), we suggested that at least two enzymes of USTB-05 participated in biodegrading NOD. The first enzyme hydrolyzed Arg-Adda peptide bond of cyclic NOD and converted it to linear NOD as the first product. The second enzyme was found to cut off the target peptide bond between Adda and Glu of linearized NOD, and Adda was produced as a second and dead-end product. This finding is very important in both basic research and the application of USTB-05 on the removal of NOD from a water environment.

## 1. Introduction

There is a drastically increasing trend for the breakout of harmful cyanobacterial blooms in recent decades, posing a serious threat to the natural ecological system. One of the biggest hazards of cyanobacterial blooms is to produce and to release various kinds of microalgal toxins, in which toxic cyclic peptides, such as microcystins (MCs) and nodularin (NOD), are most frequently detected all over the world [1]. NOD is a cyclic pentapeptide hepatotoxin produced by the filamentous cyanobacterium *Nodularia spumigena,* which is usually found in brackish water, such as the Baltic Sea [2], Lakes Alexandrina and Albert in South Australia [3], and brackish lakes or lagoons [4,5]. Furthermore, the nodularin variants (L-Har2) produced in freshwater have also been reported [6]. NOD shows a potent acute hepatotoxicity and tumor-promoting activity in domestic animals and humans by inhibition of the eukaryotic proteins phosphatase 1 and 2A [7]. Furthermore, NOD has also been proven to be not only a tumor promoter, but also a tumor initiator [8]. The LD_50_ value of NOD is approximately 50 μg/kg body weight (intraperitoneal mouse) [9]. The incidents of animal poisoning and human health problems resulted from toxic blooms of *Nodularin spumigena* have been well documented [5,10,11,12].

NOD is a compound of monocyclic pentapeptide consisting of cyclo[-d-erythro-β-methylAsp (iso-linkage)-l-Arg-Adda-d-Glu(iso-linkage)-Mdhb], where Mdhb stands for *N*-methyldehydrobutyrine and Adda is the particular C20 β-amino acid: (2*S*,3*S*,8*S*,9*S*)-3-amino-9-methoxy-2,6,8-trimethyl-10-phenyldeca-4(*E*),6(*E*)-dienoic acid. NOD closely resembles to MCs with respect to structure and biological activity [13]. There have been nine different analogues of NOD documented to date, varying usually by the variable arginine and the degree of methylation and stereochemistry [14,15]. NOD is water-soluble and chemically stable owing to its cyclic structure and the existence of several special amino acids [16], which is recalcitrant to treatments such as boiling, chemical hydrolysis and oxidation at near neutral pH [15]. Bioremediation is considered to be one of the most eagerly anticipated biotechnologies that holds promise for a successful and cost effective solution for removing NOD from water without damaging the natural environment. Some bacterial strains of *Sphingomonas* sp. [17], *Paucibacter toxinivorans* [16], and *Brevibacterium* sp. [18] have abilities to biodegrade MCs and NOD. The pathways [19], enzymes, and genes [19,20,21] which are responsible for the biodegradation of MCs, have been widely investigated, but less information was reported on the pathway for biodegrading NOD, although it is also frequently present.

In 2010, a MC-degrading bacterium designated USTB-05 was isolated from Dianchi Lake and found to be capable of biodegrading MC-RR, which was identified as *Sphingopyxis* sp. by the analysis of 16S rDNA (GenBank database under accession number: EF607053) [22]. Furthermore, MC-LR and MC-YR could also be totally removed by USTB-05 and its crude enzymes (CEs) [23,24], indicating that USTB-05 indeed possesses a strong ability in biodegrading MCs. Enzymatic pathways for biodegrading MC-RR, MC-LR, and MC-YR by USTB-05 have also been elucidated [22,23,24]. 

Here, we found that NOD could be biodegraded by both whole cells of USTB-05 and its CEs. One intermediate and one dead-end product of NOD catalyzed by CEs were observed on the profiles of HPLC with time course, indicating that at least two enzymes were involved in the biodegradation of NOD. These findings are of vital importance for both the basic study and the practical application in the removal of NOD by USTB-05 from natural water sources.

## 2. Results

### 2.1. Biodegradation of NOD by Sphingopyxis sp. USTB-05

**Figure 1:**
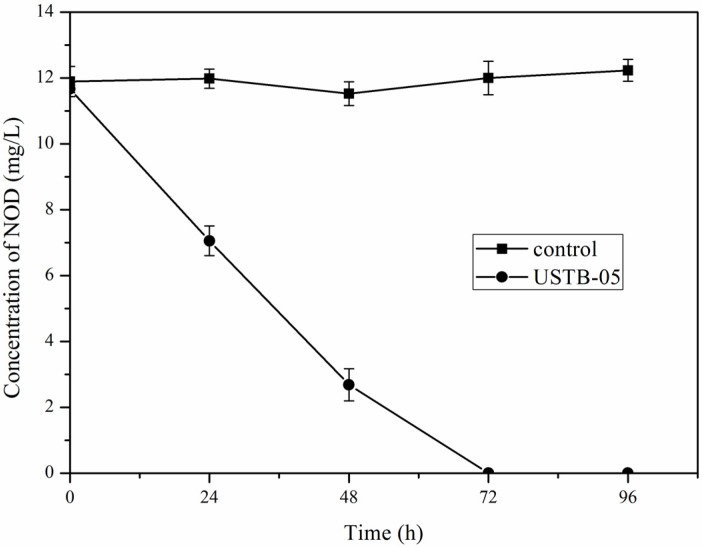
Biodegradation kinetics of NOD by whole cells of *Sphingopyxis* sp. USTB-05. Biomass of strain USTB-05 with an optical density at 600 nm (OD_600_) of 0.5 ± 0.02 was used. Standard errors are displayed (*n* = 3).

Figure 1 shows the biodegradation kinetics of NOD by USTB-05 and demonstrates that the initial concentration of 11.6 mg/L NOD was completely eliminated within 72 h while NOD in the control group remained almost constant. The average biodegradation rate of NOD was 3.9 mg/L per day, indicating that USTB-05 has a strong ability in removing NOD.

### 2.2. Enzymatic Kinetics of the NOD Biodegradation by CEs of USTB-05

**Figure 2:**
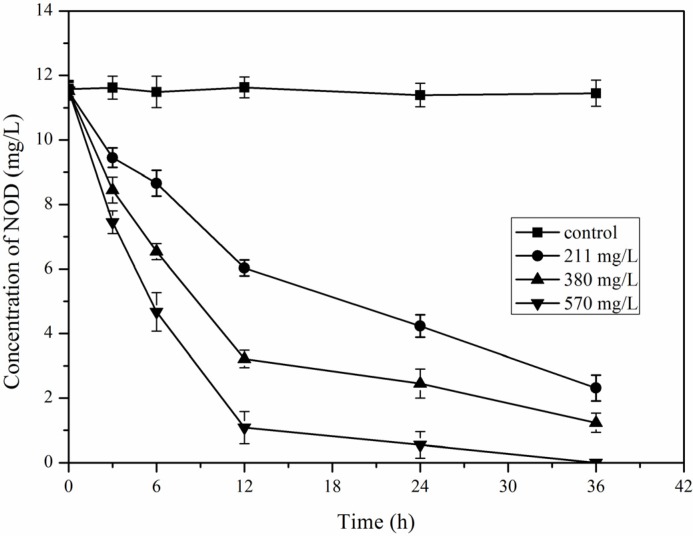
Biodegradation kinetics of NOD catalyzed by CEs containing different protein concentrations. Standard errors are displayed (*n* = 3).

Figure 2 demonstrates the biodegradation kinetics of NOD catalyzed by CEs of USTB-05 containing different protein concentrations of 211, 380 and 570 mg/L, respectively, which indicated that the removal rate of NOD was greatly improved with the increase of protein content. The initial concentration of 11.6 mg/L of NOD could becompletely eliminated within 36 h when the protein concentration was 570 mg/L, while NOD in control group remained also almost constant. Compared with the cells of USTB-05 (Figure 1), its CEs showed a higher biodegradation rate.

### 2.3. Products of NOD Detected at the Wavelength of 238 nm on HPLC

**Figure 3:**
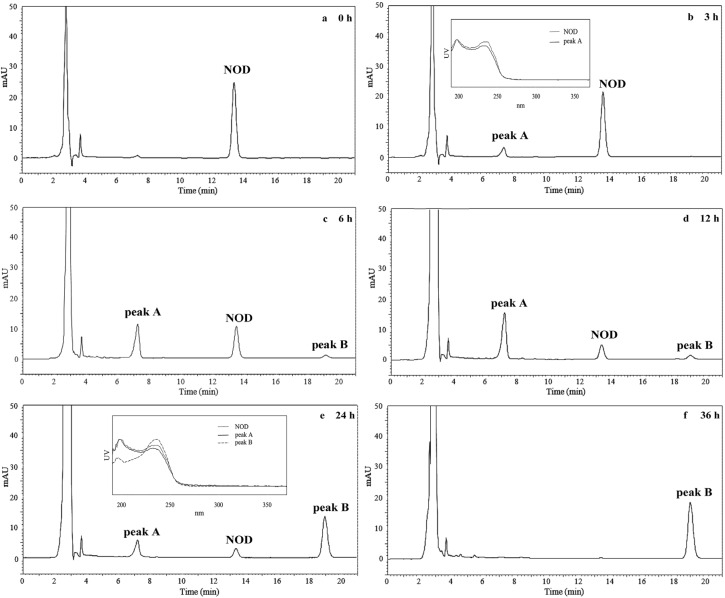
High performance liquid chromatography (HPLC) profiles for the enzymatic biodegradation of NOD by CEs after the following times: (**a**) 0 h; (**b**) 3 h; (**c**) 6 h; (**d**) 12 h; (**e**) 24 h; and (**f**) 36 h. The scanning profiles of NOD and its products at the ultraviolet wavelength from 200 nm to 370 nm are shown in (**b**,**e**).

Figure 3 shows the biodegradation products of NOD in the presence of CEs (570 mg/L protein) detected on HPLC. The peak of NOD at the retention time of 13.5 min gradually decreased over time, and completely disappeared at 36 h (Figure 3). A new peak of product A at the retention time of 7.2 min appeared at 3 h (Figure 3b), increased (from Figure 3b–d), and then decreased (Figure 3e,f). As shown in Figure 3c, another new peak of product B at the retention time of 19.2 min appeared at 6 h and always increased over time until 36 h. The ultraviolet absorption spectra of both peak A and B were very similar to that of NOD in the wavelength from 200 nm to 370 nm (Figure 3b,e) suggesting that Adda was still present in these two products. The results indicate that CEs of USTB-05 has the enzymatic activity to catalyze NOD. The first intermediate of NOD is product A (retention time of 7.2 min). Afterwards, product A is completely converted to product B (retention time of 19.2 min).

### 2.4. RRLC-MS Analysis of NOD and Its Biodegradation Products

**Figure 4:**
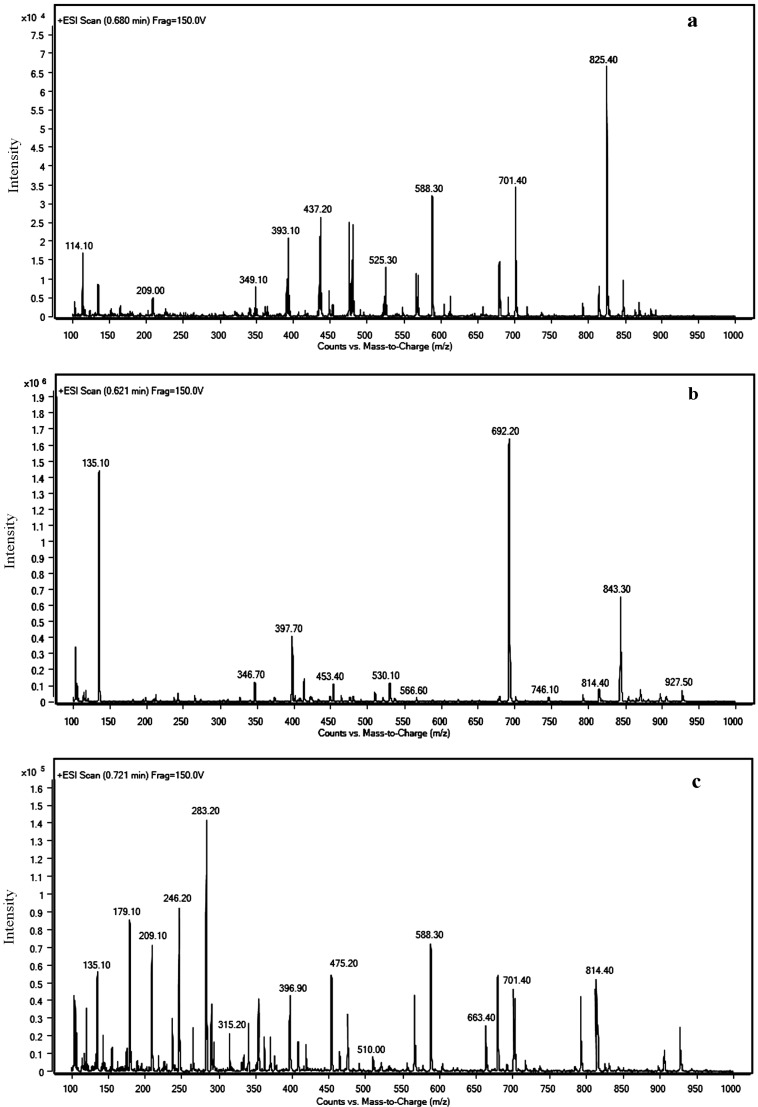
RRLC-MS profile for NOD and its biodegradation products: (**a**) MS spectrum for NOD; (**b**) MS spectrum for product A; and (**c**) MS spectrum for product B.

HPLC analysis of samples containing CEs of USTB-05 revealed the presence of two biodegradation products and showed that the absorption spectra of the two products were extremely similar to that of NOD. Based on the retention time and UV chromatogram in the HPLC profiles, peaks at the retention times of 13.5, 7.2, and 19.2 min were identified as NOD, product A, and product B, respectively. To characterize the products, RRLC-MS was used to measure the mass/charge (*m*/*z*) ratios of NOD and its two products. The mass spectral analysis of NOD showed a major ion at *m*/*z* 825.4, corresponding to the [M + H]^+^ protonated molecular ion (Figure 4a).

The mass spectrum for product A showed a protonated molecular ion at *m*/*z* 843.4 and a prominent ion at *m*/*z* 692.2 (Figure 4b). An ion peak at *m*/*z* 843.4 indicates that product A was linearized NOD [M + H_2_O + H]^+^. The major peak with *m*/*z* 692.2 [M + H_2_O − 151 + H]^+^ is *m*/*z* 151 lower than that of linearized NOD, which corresponds to the loss of terminal phenylethymethoxy group (PhCH_2_CHOCH_3_, MW: 135) and the amino NH_2_ group (MW: 16) from Adda after Arg-Adda bond breaking. This is a typical loss fragment from a linear NOD with Adda due to the radical fragmentation rearrangement [25]. The presence of this peak evidences that the linearized NOD product contained *N*-terminal Adda. Moreover, the presence of carboxy-terminal arginine (Arg) was demonstrated by protonated ion *m*/*z* 530.1 [Glu-Mdhb-MeAsp-Arg-OH + 2H]**^+^** (Figure 4b).

Figure 4c reveals that the protonated molecular ion of product B was detected at *m*/*z* 663.4, which coincides with the dimeric ion of Adda (MW: 331) [26,27]. Additionally, fragments at *m*/*z* 315.2 and *m*/*z* 135.1 are related to the loss of the amino NH2 group (MW: 16) from Adda and the PhCH2CHOCH_3_ part of Adda, respectively. Other fragment ions that support the elucidation of the structure of product B were recorded at *m*/*z* 283.2 [Adda − NH_3_ − CH_3_OH + H]^+^ and 179.1 [Adda − PhCH_2_CHOCH_3_ − NH_3_ + H]^+^. Therefore, it is inferred that product B was Adda.

## 3. Discussion

MCs and NOD are common organic contaminants in natural eutrophic lakes and reservoirs around the world and there is an urgent need for an efficient and low-cost technology to eliminate cyanobacterial toxins from drinking water. Biodegradation has been found to be one of the essential processes for removing cyanotoxins. Both a microbial community [28] and the single bacterial strain [29,30] were confirmed to be capable of biodegrading MCs. It has also been revealed that biodegradation of NOD by an indigenous bacterial community is the main pathway of this toxin’s removal [26,27,31]. However, a lag phase that lasted from two days to three weeks was present in the biodegradation process before the rapid decomposition of this toxin. It is universally known that the enzymes degrading cyanobacterial peptide toxins possess substrate-specificity and, thus, not all MC-degrading bacteria can hydrolyze peptide bonds in NOD [19,29]. So far, the number of bacterial species reported to be capable of degrading NOD is very limited [16,17,32] and less information was avaiable on NOD, although it was also frequently found in natural water body. *Sphingomonas* sp. 7CY, isolated from Lake Suwa in Japan, has been reported to biodegrade NOD-Har containing the homoarginine residue in place of arginine of NOD, but only in the presence of MC-RR [33]. Thirteen bacterial strains isolated from sediments and water of Finnish lakes [16] and five bacteria isolates from three Scottish waters [18] were found to have the abilities in biodegrading both MCs and NOD, but the degradation rates were considerably slow. Cell extract from *Sphingomonas* sp. B-9 (Lake Tsukui, Kanagawa, Japan) was also found to remove NOD, but only Adda was detected as a final product [17].

As for the biodegradation products of NOD, Kato *et al.* (2007) confirmed that NOD could be biodegraded by CEs of strain B-9 and linear NOD and Adda were identified as a degradation intermediate and a final product, respectively [32]. Mazur-Marzec *et al.* (2009) conducted regular studies on Gulf of Gdańsk, southern Baltic Sea, which experienced high blooms of toxic cyanobacteria [26]. In their study, incubation of NOD in the presence of sediments from the Gulf of Gdańsk resulted in the formation of several products of microbial degradation including a linear NOD, Adda, and two tetrapeptides: H-Adda-Glu-Mdhb-MeAsp-OH and H-Glu-Mdhb-MeAsp-Arg-OH. The results confirmed that various biodegradation pathways of NOD are possible. Based on the successful isolation and identification of *Sphingopyxis* sp. USTB-05 for the biodegradation of MC-RR [22], we have continuously investigated its genes and enzymes involved in biodegrading MC-RR, MC-LR, and MC-YR, and three biodegradation genes have been firstly cloned and expressed [24,34,35]. The first enzyme, microcystinase (coded by the gene *USTB-05-A*), can turn the highly-stable cyclic MC-LR, MC-RR or MC-YR into a linear structure by the hydrolytic cleavage between Adda and Arg. The second enzyme, serine protease (coded by the gene *USTB-05-B)*, is responsible for converting linearized MC-LR, MC-RR, or MC-YR into the tetrapeptide of NH_2_-Adda-Glu (iso)-Mdha-Ala-OH. The third enzyme, peptidase (coded by the gene *USTB-05-C*), plays a role in cleaving the tetrapeptide to produce Adda [24,34,35]. Compared with MCs, all of which contain seven amino acids, NOD is a much tighter and smaller ring with only five amino acids and it has been indicated that this makes it harder to open the ring structure to facilitate degradation [18]. According to our study, an initial 11.6 mg/L of NOD was completely eliminated by USTB-05 and its CEs within 72 h and 36 h, respectively (Figure 1 and Figure 2). In order to investigate the biodegradation pathway of NOD, Adda with the conjugated diene in NOD was traced, and two biodegradation products were successfully observed with the time course at the wavelength of 238 nm on the HPLC profiles (Figure 3). Both products characterized by the UV absorption spectra were similar to NOD.

**Figure 5:**
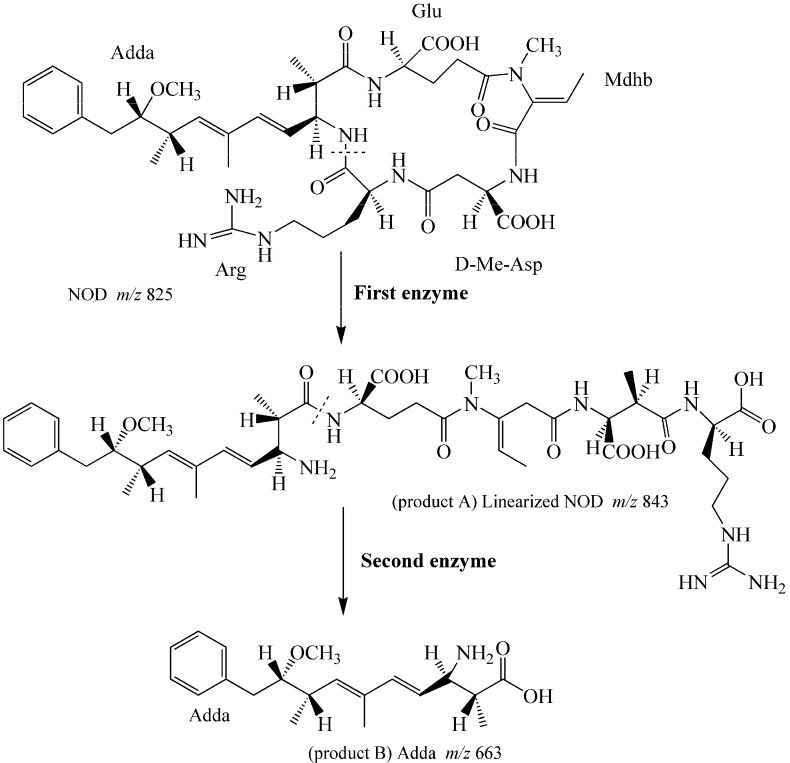
Proposed biodegradation pathway of NOD by *Sphingopyxis* sp. USTB-05.

Successful analysis of mass/charge (*m*/*z*) ratio of NOD and its two products by RRLC-MS provided evidences for the biodegradation pathway of NOD by *Sphingopyxis* sp. USTB-05 (Figure 4). The first enzyme was active in cleaving the target peptide bond between Arg and Adda of NOD (*m*/*z* 825) and converting cyclic NOD to linear NOD (NH_2_-Adda-Glu-Mdhb-MeAsp-Arg-OH) (*m*/*z* 843, product A) as the intermediate product. Then, the second enzyme was found to cut off the target peptide bond between Adda and Glu of linearized NOD, producing Adda (*m*/*z* 663, product B) as the final product (Figure 5). Generally, the biodegradation pathway of NOD by USTB-05 was similar to that of MC-RR, LR, and YR, as we have reported previously [14,22,23,24]. These findings might provide valuable evidence for further investigation on the biodegradation mechanism of NOD and its analogues by *Sphingopyxis* sp. USTB-05.

## 4. Conclusions

Based on the isolation of the promising bacterial strain *Sphingopyxis* sp. USTB-05 for the biodegradation of MCs, we successfully investigated its enzymatic biodegradation pathway on NOD. Firstly, an initial NOD amount of 11.6 mg/L was completely biodegraded within 72 h and 36 h by USTB-05 and its CEs containing 570 mg/L protein, respectively. Secondly, two products of NOD were observed on HPLC profiles at the wavelength of 238 nm during the biodegradation of NOD catalyzed by CEs. Both NOD and its two products were found to have similar UV scanning profiles at wavelengths from 200 to 370 nm, indicating that the group of Adda in products of NOD remained intact. Thirdly, the enzymatic biodegradation pathway of NOD by *Sphingopyxis* sp. USTB-05 was suggested. The first enzyme was active in cleaving the target peptide bond between Arg and Adda of NOD and converting cyclic NOD to linear NOD as the first product. Then the second enzyme was found to cut off the target peptide bond between Adda and Glu of linearized NOD resulting in the formation of Adda as the final product. This study is crucial, not only basic research, but also as a promising approach to remove NOD in future water treatment strategies.

## 5. Materials and Methods

### 5.1. Chemicals

Standard NOD (≥95% purity, molecular formula: C_41_H_60_N_8_O_10_, MW: 824, Enzo Science Inc., New York, NY, USA) of 500 μg was dissolved in deionized water at a 100 mg/L concentration and stored at −20 °C until use. Chromatographic grade acetonitrile was used to prepare the mobile phase in HPLC and RRLC-MS analyses. All other chemicals used in this study were analytical grade except those specified by the kits.

### 5.2. Bacterial Strain and Cultural Conditions

A promising bacterial strain of *Sphingopyxis* sp. USTB-05 was used as previously described [22]. The culture medium was used as mentioned in the previous report [22]. The prepared medium was sterilized at 121 °C for 20 min and then the medium was inoculated with USTB-05. The bacterial strain was cultured at 30 ^°^C and 200 r/min.

### 5.3. Preparation for the Crude Enzymes (CEs) of Sphingopyxis sp. USTB-05

Bacterial cells of USTB-05 in the logarithmic growth phase of the third day were harvested by centrifugation at 8000 r/min for 20 min at room temperature. The bacterial pellet was subsequently washed three times with 50 mM phosphate buffer solution (PBS, pH 7.0). Afterwards, the cells were resuspended in 30 mL PBS, and then disrupted using an ultrasonic disruptor with an output power of 600 W for 15 min in an ice bath. The cell debris was removed by centrifugation (13,000 r/min, 30 min, 4 °C) and the supernatant was collected and used as CEs for the enzymatic biodegradation of NOD. The concentration of protein in CEs was 760 mg/L determined with the Bradford Protein Quantification Kit (Beijing Solarbio Science and Technology Co., Ltd., Beijing, China).

### 5.4. NOD-Biodegrading Activity of Sphingopyxis sp. USTB-05

The bacterial cells of USTB-05 in the logarithmic growth phase of the third day (OD_600_ = 1.0 ± 0.05) were harvested by centrifugation (8000 r/min, 15 min), washed three times with 50 mM PBS (pH 7.0), and resuspended into 2 mL mineral salt medium [22] containing standard NOD (11.6 mg/L) in a 10 mL triangle bottle. The initial OD_600_ was adjusted to 0.5 ± 0.02. The reaction was performed at 30 °C and 200 r/min. Samples of 300 μL for each were taken after 0, 24, 48, 72, and 96 h, respectively. The control bottle was prepared in the same way without bacterial cells. All samples were centrifuged at 13,000 r/min for 10 min and then the concentration of residual NOD in supernatants was determined by HPLC. All experiments were carried out in triplicate.

### 5.5. Enzymatic Biodegradation of NOD

The total biodegradation reaction volume was 5 mL containing 570 mg/L, 380 mg/L, and 211 mg/L proteins in PBS in each 10 mL centrifuge tube, respectively. NOD was added to final concentration of 11.6 mg/L for each. The reaction was performed at 30 °C and 200 r/min. Samples of 500 μL for each were taken after 0, 3, 6, 12, 24, and 36 h, respectively, and then 5 μL of concentrated hydrochloric acid (36%, *v*/*v*) was added to stop the reaction. A control bottle was prepared in the same way without CEs. All samples were centrifuged at 13,000 r/min for 10 min and then the concentrations of residual NOD and its products in supernatants were analyzed using HPLC. All experiments were carried out in triplicate.

In order to identify the degradation products of NOD, samples of 1 mL were passed through a C_18_ solid-phase extraction cartridge (Waters, Oasis™ HLB, Milford, MA, USA, 30 mg/mL) that had been activated and equilibrated with 1 mL methanol and 1 mL water. 1 mL of water was used to remove impurities and then 1 mL methanol was used to elute NOD and its products at the rate of 1 mL/min. The elution was used to measure the mass/charge (*m*/*z*) of NOD and its products with rapid resolution liquid chromatogram-mass spectrum (RRLC-MS).

### 5.6. Analysis of NOD and Its Biodegradation Products

NOD and its enzymatic biodegradation products were firstly analyzed using a HPLC system (Shimadzu LC-10ATVP, Shimadzu Co., Ltd., Kyoto, Japan) with an ultraviolet (UV) Diode Array Detector at 238 nm using a Agilent TC-C_18_ column (4.6 mm × 250 mm) (Agilent Technologies Inc., 1200 series, Santa Clara, CA, USA). The mobile phase was water containing 0.05% (*v*/*v*) of trifluoroacetic acid (A) and acetonitrile (B) at a ratio of 65:35 (*v*/*v*). The flow rate was 1 mL/min and the injection amount was 20 μL. The concentration of NOD was determined by calibration of peak area (at 238 nm) with that of the standard NOD. The biodegradation products that contained Adda were monitored by HPLC based on the assumption that their molar absorption coefficient at 238 nm is the same as NOD.

Mass spectral analysis of the products were performed using a RRLC-MS. The RRLC system consist of an Agilent 1260 Infinity Binary pump, a 1260 autosampler, and a column compartment (Agilent Technologies Inc., Santa Clara, CA, USA). The detector used was an Agilent 6420 Triple Quadrupole mass spectrometer equipped with an electrospray ionization source operating in positive ionization mode. The analytical column was a ZORBAX Rapid Resolution SB-C_18_ column (50 mm × 2.1 mm i.d., 1.8 μm, Agilent) maintained at 30 °C. The mobile phase for RRLC-MS analysss was a mixture of water with 0.1% formic acid and acetonitrile (40:60, *v*/*v*) flowing at 0.4 mL/min. The injection volume was 5 μL. The MS detection conditions used were a desolvation gas temperature of 300 °C, desolvation gas flow of 10 L/min, nebulizer gas (N_2_) pressure of 35 psi, and capillary voltage of 4000 V.
